# Interventions targeting children and young people’s physical activity behavior at home: A systematic review

**DOI:** 10.1371/journal.pone.0289831

**Published:** 2023-08-09

**Authors:** Amanda L. Seims, Jennifer Hall, Daniel D. Bingham, Amy Creaser, Anastasia Christoforou, Sally Barber, Andy Daly-Smith

**Affiliations:** 1 Bradford Institute for Health Research, Bradford Teaching Hospitals NHS Foundation Trust, Bradford Royal Infirmary, Bradford, West Yorkshire, United Kingdom; 2 Faculty of Health Studies, University of Bradford, Bradford, West Yorkshire, United Kingdom; 3 Wolfson Centre for Applied Health Research, Bradford Royal Infirmary, Bradford, West Yorkshire, United Kingdom; 4 Department of Strategy, Research & Impact, UK Youth, London, United Kingdom; Mid Sweden University: Mittuniversitetet, SWEDEN

## Abstract

**Background and purpose:**

Increased time at home during the COVID-19 pandemic significantly decreased children’s physical activity. This systematic review aimed to evaluate the effectiveness of children’s home-based physical activity interventions, and identify ‘active ingredients’ underpinning these.

**Methods:**

Databases searched—AMED, PsychINFO, CINAHL, Cochrane, EMBASE, PubMed/Medline, Scopus, SPORTDiscus and Web of Science, from inception until June 2022. Eligibility criteria–children aged 2–16 years, targeting home-based physical activity, a control group, and physical activity measured pre- and post- intervention. Studies were excluded if it was not possible to identify change in physical activity at home. The review was written following the Preferred Reporting Items for Systematic Reviews and Meta-Analyses (PRISMA) guidance. Study quality was evaluated using the quality assessment tool for quantitative studies. Study design, intervention characteristics, outcome data, behavior change theory, Behavior Change Techniques (BCTs) and process evaluation data were extracted and discussed using narrative syntheses.

**Results:**

13 studies (including 1,182 participants) from 25,967 were included. Interventions primarily involved active video games, with the addition of coaching or telehealth support (n = 5). Three of the 13 studies significantly increased children’s physical activity (1 = Moderate to vigorous physical activity, 2 = total volume, *P*<0.05). The largest effect size (d = 3.45) was for moderate to vigorous physical activity. 29% of BCTs were identified across included interventions; the most common being adding objects to the environment. The most effective intervention scored strong for design quality, incorporated telehealth coaching, and included the most commonly coded BCTs. Variation among studies and insufficient reporting of data made a meta-analysis unfeasible.

**Conclusion:**

COVID-19 emphasized the importance of the home for physical activity. Whilst effectiveness of interventions was limited, building social support and self-efficacy are mechanisms that should be explored further. The review provides recommendations to improve the design and evaluation of future interventions.

**Trial registration:**

Prospero registration number: CRD42020193110.

## Introduction

Only 45% of children and young people (CYP) (5–16 years) in England engage in the recommended daily average of 60 minutes of moderate to vigorous physical activity (MVPA) [1, 2]. Typically, children spend almost half of their time at home [3, 4], yet, only a small amount of this time is spent active [3]. Due to such low levels of physical activity, CYP are not gaining the multitude of benefits from leading an active lifestyle such as improved psychological wellbeing, academic attainment and physical health [5, 6].

The national UK lockdown in response to the COVID-19 pandemic advised people to ‘stay at home’, limiting physical activity opportunities. In response, there was a rapid mobilization of online physical activity opportunities targeted at CYP [7–9], and renewed research interest in the home environment [10]. Despite such opportunities, children’s total and relative time spent in MVPA at home [11], and overall MVPA [12] significantly decreased.

Understanding effective approaches to enhance CYP’s physical activity at home is important for minimizing the impact of future pandemics through informing policies and practice [13]. However even under normal circumstances, it is essential to support children potentially struggling to access physical activity opportunities beyond the home. Such children include those with health conditions or disabilities that limit movement, or who provide care for family members. It is also particularly important for children living in neighborhoods where parents fear for their child’s safety [14], and younger children who, compared to older children, have less independent mobility [3].

Activating inside space is vital for children who live in homes with no garden. This is pertinent for children from minority ethnic groups, as ONS [15] data shows 1 in 8 British households have no garden, with Black people 4 times as likely as White people to have no access to outdoor space. Outside of single-sex physical education, minority ethnic young women reported engaging in little or no physical activity in public spaces, often using bedrooms, gardens and living rooms, which provided safe and intimate spaces for movement [16]. The home is an important setting for South Asian Muslim adolescent girls’ experiences of physical activity away from school, providing a private and safe space to feel relaxed and explore their physicality [17].

Previous reviews of CYP’s home-based interventions often describe an intervention focused on family support that influences physical activity in all environments, not just the home [18–20]. To our knowledge, only 2 systematic reviews [21, 22] have included interventions specifically targeting young people’s physical activity at home.

Maitland *et al’s*. [21] review included 3 randomized controlled trials using active video games (AVGs), with 1 significantly increasing physical activity [23]. The review by Kaushal and Rhodes [22] was not solely focused on children, however it included 3 child-focused randomized controlled trials in addition to those included in the previous review [21]. All studies included AVGs, with only 1 showing a group effect but no interaction [24]. Both reviews primarily reported adherence, with limited discussion of effects on MVPA. This, along with the limited number of effective studies focused on CYP, makes it difficult to identify mechanisms of effectiveness and understand how we can increase CYP’s physical activity at home.

Behavioral change theories attempt to explain the process of how human behaviors change. Behavior change techniques have been defined as irreducible, observable, and replicable components of an intervention designed to redirect behavior [25]. The behavior change technique taxonomy [26], provides a standardized system for classifying intervention components. Identifying behavior change techniques in addition to intervention characteristics, could help to identify potential mechanisms of action. Neither of the previous reviews [21, 22] explored behavior change theories underpinning interventions or characterized the interventions using the behavior change technique taxonomy, which may have facilitated understanding of why some interventions were effective and some were not.

The purpose of this systematic review was to understand the effectiveness of interventions on young people’s physical activity in the home environment. Furthermore, it aimed to identify the characteristics of the successful interventions and the behavior change techniques underpinning these, to provide recommendations for the design of future interventions targeting children’s physical activity at home.

## Methods

The Preferred Reporting Items for Systematic Reviews and Meta-Analyses (PRISMA) [27] was followed and the protocol published on PROSPERO (20/8/20; CRD42020193110, https://www.crd.york.ac.uk/prospero/display_record.php?RecordID=193110). Since registration, the protocol was amended to include process evaluation data within the analysis to support the identification of mechanisms of effectiveness.

### Eligibility criteria

PICOS (population, intervention, comparator, outcome, study design) was used to establish inclusion criteria [28]:

- Population: CYP aged 2–16 years (or a mean age within that range) through direct or family-based interventions. A broad age range to include early years and adolescents was chosen to maximize the number of studies for the review, given that previously published reviews primarily reported studies using adults.
- Intervention: Targeting CYP’s physical activity behavior at home (inside or within the immediate vicinity of the home). No restrictions on intervention approach.
- Comparator: Control group (no treatment), wait-list control or an alternative intervention.
- Outcome: Device-based (e.g. accelerometer) or self-report (e.g. child or parent questionnaire) or change in minutes of CYP physical activity levels.
- Study design: Pre-post control design, including randomized and quasi control trials.

Where the home was the sole focus of the intervention, changes in physical activity were assumed to reflect home-based behavior change, as no other behavior was targeted. Interventions targeting the home within wider initiatives were excluded unless physical activity outcome data could distinguish between the home and other settings. Studies were excluded where interventions primarily targeted physical activity at locations outside the home, or lacked a comparator group. Exclusion criteria extended to review or discussion articles, not full articles published in a peer-reviewed journal (e.g. conference abstracts, commentaries), PhD thesis, grey literature, and non-English language articles.

Although the aim of the review was to identify behavior change techniques used in interventions, the use of behavior change techniques was not part of the eligibility criteria for inclusion.

### Search strategy

Electronic databases searched included: AMED, PsychINFO, CINAHL, Cochrane, EMBASE, PubMed/Medline, Scopus, SPORTDiscus and Web of Science, from inception until June 2022. Dates of searches, coverage dates, and search criteria adapted for each database can be viewed in S1 Table. The search criteria included the following terms:

- Population = Child*, young person, adolescence, adolescents, teen*, girl*, boy*, infant*, family, toddler AND
- Setting = Home*, community, fam AND
- Target behavior = physical activity, exercise, fitness, play, move, dance, sport AND
- Outcome = step count, accelerometer, pedometer, GPS, global positioning system, moderate to vigorous physical activity, MVPA, minutes AND
- Intervention = intervention, toolkit OR resource OR campaign OR promotion OR trial or “randomi*ed controlled trial” OR “controlled trial” OR RCT OR “primary prevention” OR strategy OR program* OR experiment* OR quasi

Reference lists of included papers were searched for additional studies meeting the inclusion criteria.

### Selection process

The lead author (AS) removed duplicate records and imported records retrieved into Microsoft Excel (Microsoft Corporation, 2010). Researchers (MW, IS, DM and EY) worked in pairs, and each pair independently reviewed article titles and abstracts. Titles were excluded if deemed irrelevant subject matter or where exclusion criteria were met. Abstracts were excluded if they were a conference abstract only, online thesis, or if at least 1 of the inclusion criteria was not met. Where agreement within pairs could not be reached, abstracts were reviewed by AS, ACh, JH or ZK. Researchers (MW, IS, DM and EY) worked in pairs to independently review all the full papers. All full papers were third reviewed by AS and ACr and excluded if at least 1 of the inclusion criteria were not met.

### Data collection process

AS and ACh independently extracted data from included studies, then reviewed together to discuss discrepancies and reach a consensus on the level of information for inclusion. Protocol papers referred to within selected studies were also used to obtain data and provide clarification. Key study characteristics included participants, country, design, setting, outcome measure, effectiveness. Device-based measurement of physical activity outcome data was prioritized where more than 1 instrument was used (e.g. accelerometer and self-report). To identify how and why studies were/were not effective, process evaluation data within included studies (including those published separately) and intervention implementation characteristics (delivery mode [including the person/people delivering the intervention], duration and frequency, and engagement and compliance measures [including outcomes if reported sufficiently]) were extracted. Behavior change theory was also noted where mentioned by the authors, to identify the theory informing the intervention.

### Behavior change technique coding

ACr and JH used the behavior change technique (BCT) taxonomy coding framework [26] to ascertain which BCTs were present within interventions. BCTs were coded as either “present beyond all reasonable doubt” (++) or “present in all probability” (+) as recommended [26]. BCTs were coded within source papers descriptions of intervention and control/comparator groups, and where they were applied to encourage parental support behavior; the target behavior being supporting and encouraging their children to be physically active. Reviewers met to discuss the coding, and any disagreements were resolved through discussion.

### Quality of studies assessment

AS and ACh independently reviewed the selected articles for quality [29]. Discrepancies were discussed until a consensus was reached. The scoring criteria used a rating of ‘weak’, ‘moderate’ or ‘strong’ for the study components including: selection bias, design, confounders, blinding, data collection methods, and withdrawals and drop-outs. The overall paper rating was classed as ‘strong’, where no components were rated as ‘weak’; ‘moderate’ if 1 ‘weak’ component; and ‘weak’ if two or more ‘weak’ components.

### Data synthesis

The key study variables and group descriptive statistics (including Cohen’s *d* effect size where feasible) were synthesized in a table, with studies themed according to the intervention mode. A narrative synthesis was used to compare and contrast each extracted element of the study and intervention characteristics. Recruitment rate was calculated as the proportion of participants randomized, out of those deemed eligible (where reported). Retention rate was calculated as the proportion of participants completing outcome measures, out of those randomized. Intervention characteristics were synthesized in a separate table. A narrative summary of process evaluation data was used to describe the barriers and facilitators to adherence to the interventions. Heterogeneity of studies made a meta-analysis impractical.

## Results

**Fig 1:**
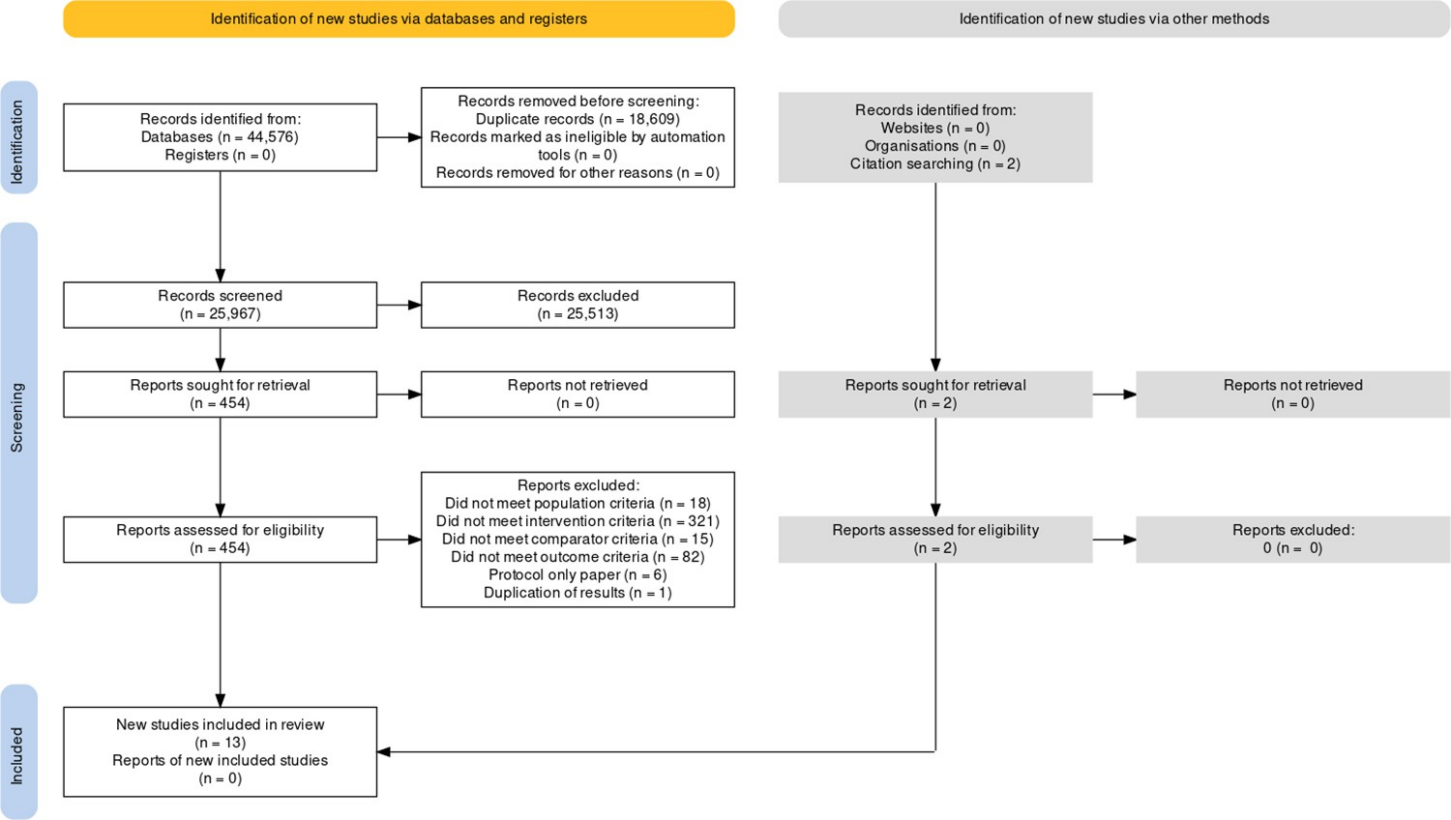
PRISMA (2020) Flow chart of data extraction process. (Image created at https://www.eshackathon.org/software/PRISMA2020.html).

Including duplicates, 37,477 articles were retrieved in the first search, and an additional 7,099 through subsequent searches (Fig 1 and S1 Table).

When reviewing full articles, a further 5 studies initially appeared to meet the inclusion criteria [30–34], but were subsequently excluded for the reasons stated (S2 Table).

Overall, 13 studies were included with 7 new contributions since previous reviews [21, 22]. Interventions were most commonly implemented in the USA (5/13 studies), with the rest in Australia, New Zealand, Canada, UK and Finland. Interventions traditionally incorporated active video games (AVGs), but more recent interventions included the addition of telehealth coaching and/or physical activity equipment. Only 3 of the 13 studies significantly increased physical activity. A wide range of behavior change techniques (BCTs) were identified within all included studies, with ‘adding objects to the environment’ common across all 3 effective studies.

### Study and intervention characteristics

Data are presented in Tables 1 and 2.

**Table 1:** Study characteristics, outcomes and effects.

Study	Country		Participant characteristics sample size; age in years (range/mean); % female; % ethnicity	Study design	Intervention setting and mode	Recruitment (Rec) & retention (Ret) rate	PA Outcome measure	Intervention effects
**AVGs only interventions**								
Baranowski *et al*. [37]		USA	Children at risk for adult obesity; n = 78 (41 intervention and 37 control); 11.3 years; 49% F; 41% African American, 14% White, 13% Hispanic, 28% mixed ethnicity, 4% other	RCT—intervention and control groups	Home-based AVGs vs inactive video games	Rec—not reported Ret—92%	CPM and time in LPA and MVPA by 7 day hip-worn accelerometry (ActiGraph GT3) baseline, week 1, 6, 7 & end.	No significant interaction for PA. MVPA Mean differences—Baseline: 2.35 min^.^d^-1^ (95% CI 25.32 to 10.02); End: 0.36 min^.^d^-1^ (95% CI, 26.31 to 7.03). *P* > 0.05
Graves *et al*. [36]		UK	Children who played games consoles for > = 2h per week; n = 58 (n = 29 intervention, n = 29 control); 9.2 years 0.5; 33% F	RCT—intervention and control groups	Home-based step-powered AVGs vs usual video gaming behavior	Rec—98% Ret—72%	CPM and time in PA at various predefined thresholds by 7 day hip-worn accelerometry (ActiGraph GT1M), time in step-powered gaming and AVG at baseline, mid and end intervention	No significant interaction for PA. PA_>4_ group difference = 0.6 min^.^hr^-1^ (95% CI, -2.2–1.0). *P* > 0.05
Howie *et al*. [38]		Australia	Children with developmental coordination disorder; n = 21; 11.0 years; 52% F	RCT—crossover design	Home-based AVGs vs. no AVGs	Rec—100% Ret—100%	Time in LPA, MPA and VPA by 7 day hip-worn accelerometry (Actical Respironics) and self-reported PA at baseline and in the final week.	No significant interaction for PA. MPA group difference = 0.7 (95% CI, -4.6–3.3). *P* = 0.73. *d* = -0.259 Self-reported PA increase in predominantly outdoor activities but significant reduction in outdoor play at weekends (9.8 min^.^d^-1^).
Maddison *et al*. [35]		New Zealand	Children with overweight or obesity who owned games consoles; n = 322 (n = 160 for intervention & control); 11.6 years; 27% F; 57% NZ European, 26% Pacific, 17% Maori	RCT—intervention and control groups	Home-based AVGs vs no intervention (usual video gaming behavior)	Rec—100% Ret—80%	CPM and time in LPA and MVPA—7 day hip-worn accelerometry (ActiGraph AM7) at pre, mid and end intervention.	No significant interaction for PA. MVPA group difference = 1.65 min^.^d^-1^ (95% CI, -5.77–9.07). *P* = 0.66
Mark and Rhodes [24]		Canada	n = 38 children from 60 families (equal between groups); ~5 years; 42% F	RCT—main intervention and alternative intervention	Home-based game bike (cycle-powered AVGs) vs. stationary bike in front of TV	Rec—100% Ret—90%	Self-reported log of duration and weekly frequency of bike usage	No group x time interaction for usage and duration (mean data not provided). Significant group effect with greater bike frequency and duration of usage with game bike vs. bike with TV (no time interaction) but at week 6 only (mean data not provided, *P*<0.05; *d*>0.7).
Ni Mhurchu *et al*. [23]		New Zealand	n = 20 who owned games consoles and played inactive games (10, intervention, 10 control); 12 years; 40% F	RCT—intervention and control groups	Home-based AVGs vs usual game play	Rec—not reported Ret—100%	Time in LPA, MPA and VPA—4 day hip-worn accelerometry (AM7) and self-reported (PAQ-C) at baseline, mid and end.	Significant group x time interaction for CPM, but only at 6 weeks. Group difference at 6 weeks = 194 CPM (95% CI, 32–310] *P* = 0.04); 12 weeks = 48 CPM (95% CI, -153-187). *P* = 0. No effect on MVPA or self-reported activity (mean data not provided).
Rhodes *et al*. [40]		Canada	Inactive children; n = 73 (n = 39 intervention, n = 34 control); 11.5 years; equal gender split; ~87% white.	RCT—main intervention and alternative intervention	Home-based exergame bike (cycle-powered AVGs) vs. stationary bike in front of TV	Rec—100% Ret—92%	Self-reported log of duration and weekly frequency of bike usage.	Significantly group x time interaction, with greater minutes of bike usage with exergame bike vs. bike with TV (*P*<0.05). Week 1 = 74.4 vs. 41.6 min^.^wk^-1^ (*d* = 0.52); weeks 3–9 = 19.7 vs. 14.9 min^.^wk^-1^ (*d* = 0.3) End = 7.82 vs. 6.37 min^.^wk^-1^ (*d* = 0.08). No group effect for frequency of usage, but a significant decline over time (*P* < 0.01).
**AVGs plus other component interventions**								
Errickson *et al*. [41].	USA		n = 61 (18 standard intervention, 22 intervention plus coaching, 20 control); mean age range 7.4–7.6 years; 40–55% F; 62–72% white.	RCT—2 x intervention and wait-list control groups	IG1: Home-based AVGs vs IG2: AVGs plus coaching vs no intervention	Rec—100% Ret—100%	Time in MPA and VPA by 7 day accelerometry (ActiGraph) at week 1 & end intervention.	No significant effects for any PA. MPA baseline = 140.5 min^.^d^-1^ (IG1) vs.154.6 min^.^d^-1^ (IG2) vs.116.4 min^.^d^-1^ (control); MPA end = 146.4 min^.^d^-1^ (IG1) vs.148.1 min^.^d^-1^ (IG2) vs.112.1 min^.^d^-1^ (control).
Maloney *et al*. [42]	USA		n = 60 (20 standard intervention, 20 intervention plus coaching, 20 wait-list control); 7.5 years, 50% F, 30% white	RCT—2 x intervention* and wait-list control groups	IG1: Home-based AVGs vs IG2: AVGs plus coaching vs no intervention	Rec—can’t tell Ret—85%	Minutes per day in LPA, MPA and VPA by 7 day accelerometry (ActiGraph) and self-reported PA at week 1, end and 14 week follow-up	No significant group x time interaction (*P* = 0.89). MPA change = -7.2 ± 28.3 min^.^d^-1^ (IG) vs. -4.3 ± 34.3 min^.^d^-1^ (control). Significant increase in VPA for IG (from 10.0 ± 7.7 min^.^d^-1^ to 16.2 ± 11.8 min^.^d^-1^, *P* < 0.01) which remained at 18 week follow-up (*P* < 0.01). Significant increase in MPA for waitlist control following AVGs (from 112.1 ± 36.7 min^.^d^-1^ to 135.9 ± 31.4 min^.^d^-1^, *P* < 0.005).
Mitchell *et al*. [43]	Australia		Children with unilateral cerebral palsy; n = 91 (51 intervention & 50 control); 11.3 years; 49% F	RCT—intervention and control groups	Home-based AVGs and social support vs no intervention	Rec—57% Ret—89%	CPM, step count and time in LPA and MVPA—4-day hip-worn accelerometry (ActiGraph GT3X+) baseline and end intervention.	No significant interaction for PA. Group difference = -0.1 h (95% CI, -0.2–0.1), *P* = 0.66.
Rubin *et al*. [45]	USA		n = 111; (n = 45 with Prader-Willi syndrome; n = 34 intervention, n = 11 control); (n = 66 non-syndromal obesity; n = 43 intervention and n = 23 control); mean age range from 9 to 11; ~46% F; 40% white, 41% Hispanic	Quasi-experimental (semi-random)—intervention & wait-list control groups	Home-based parent-led physical activity curriculum including AVGs and non-video-based games vs.no intervention	Rec—100% Ret—89%	Time in LPA, MPA, VPA, MVPA and TPA—8 day accelerometry (ActiGraph GT3X+) at baseline and end	No significant interaction for PA. Baseline = 39.6 min^.^d^-1^ (IG) vs. 40.6 min^.^d^-1^ (control); end = 38.9 min^.^d^-1^ (IG) vs. 38.3 min^.^d^-1^ (control); *P*>0.05.
Staiano *et al*. [47]	USA		Children with overweight or obesity; n = 46 (split equally between intervention and control groups); 11.2 years; 46% F; 57% African American; 41% White, 2% other;	RCT—intervention and control groups	Home-based AVGs with social support vs. no intervention	Rec—72% Ret = 98%	Time in MVPA—7 day accelerometer (ActiGraph GT3X+) pre and end.	Significant group x time interaction for MVPA. Group difference = 3.6 ± 3.4 min^.^d^-1^ (IG) vs. −7.8 ± 3.2 min^.^d^-1^ (control), *P* = 0.028. *d* = 3.452.
**Non-AVG interventions**								
Tuominen *et al*. [48]	Finland		n = 203 mother-child pairs (n = 101 intervention, n = 102 control); children = 6.5 years; ~50% F	RCT—intervention and control groups	Home-based movement to music	Rec—76% Ret—81%	Time in MVPA (proportion of measurement time) - 7 day accelerometer (Hookie AM20) and self-reported PA at pre and end	No significant interaction for PA. Group difference = 0.006 (95% CI, -0.016 to 0.028), *p* = 0.565.

AVGs = Active video games; CPM = counts per minute; *d* = Cohen’s *d* effect size; LPA = light physical activity; MPA = moderate physical activity; MVPA = moderate to vigorous physical activity; PA_>4_ = minutes of physical activity above 4km^.^h^-1^; RCT = randomized controlled trial; VPA = vigorous physical activity.

AVGs = Active video games; CPM = counts per minute; *d* = Cohen’s *d* effect size; IG1 = intervention group 1; IG2 = intervention group 2; LPA = light physical activity; MPA = moderate physical activity; MVPA = moderate to vigorous physical activity; PAQ-C = physical activity questionnaire—children’s; RCT = randomized controlled trial; VPA = vigorous physical activity.

AVGs = Active video games; CPM = counts per minute; *d* = Cohen’s *d* effect size; IG1 = intervention group 1; IG2 = intervention group 2; LPA = light physical activity; MPA = moderate physical activity; MVPA = moderate to vigorous physical activity; RCT = randomized controlled trial; VPA = vigorous physical activity.

* Data for the 2 intervention groups was not reported separately

MVPA = moderate to vigorous physical activity; RCT = randomized controlled trial.

**Table 2:** Intervention characteristics.

Study	Intervention delivery	Intervention duration and frequency	Engagement and compliance
**AVGs only interventions**			
Baranowski *et al*. [37]	Provided with Wii console, & peripherals. Choice of 1 AVG from a selection of 5 at week 1 & 7. Child-led engagement with intervention.	13 weeks. No prescribed game play duration or frequency.	Console log recorded date, name and duration of game play. Children & parents recorded name of game, time of day played and who played it (only during weeks 1, 6, 7 and 12).
Graves *et al*. [36]	Provided with 2 sets of pedometers and a device (jOG, New Concept Gaming Ltd) that linked to the child’s PlayStation 2/3 console controller and translated stepping action into the movement of game characters. Parent- and child-led	12 weeks, no prescribed duration or frequency of play, but participants and parents were encouraged to use the jOG setup instead of usual seated inactive game play	Self-reported use of intervention at 6 and 12 weeks. Time spent on AVGs increased significantly at mid intervention compared to control (0.95 h^.^d^-1^). Significant decrease in time spent on step-powered AVGs at end vs mid intervention (*P* = 0.01).
Howie *et al*. [38]	Provided with a Playstation3 and Move and Eye input devices, an Xbox 360 with Kinect input and 11 AVGs (additional 2 games mid intervention). Child-led where children could select the AVGs from the range provided. Researchers provided technical support every 2 weeks.	16 weeks. Requested to complete 20 minutes a day on most days of the week.	Self-reported (daily calendar) game use, physical activity and other electronic game use. Research assistants checked self-report data every 2 weeks. Mean AVG play of 140.3 ± 62.9 min^.^wk^-1^. 90% of participants met the minimum recommended average of 80 minutes per week.
Maddison *et al*. [35]	Provided with Sony Eye Toy, camera, dance mat and selection of AVGs throughout the intervention. Child-led.	24 weeks. Instructed to play AVGs to replace inactive periods and periods of playing inactive video games. Encouraged to meet 60 mins MVPA on most days	Self-reported use of intervention at 12 and 24 weeks only. Mean AVG play of 15.5 ± 26.3 min^.^d^-1^ at 12 weeks and 10.2 ± 23.9 min^.^d^-1^ at 24 weeks (<30% compliance with recommended daily game play) Significant increase of 10 min^.^d^-1^ in time spent playing AVGs l (*P*<0.01).
Mark and Rhodes [24]	Provided with a gamebike (Cat Eye Electronics) that could control games on a PlayStation 2 (they were loaned a PlayStation if they didn’t have one) and 3 games. Child-led.	6 weeks, given a copy of Canada’s PA Guide to help specify the amount of PA	Self-reported logs of duration and frequency of use. Data not provided.
Ni Mhurchu *et al*. [23]	Provided with AVGs, EyeToy and dance mat. Parent- and child-led.	12 weeks. Instructed to substitute usual non-active video game play with active game play.	Self-reported logs of active and inactive game play at baseline, week 6 and 12. Mean AVG play of 41 min^.^d^-1^. Significantly greater AVG play of 41 vs. 27 minutes (*P* = 0.03)
Rhodes *et al*. [40]	Provided with exergame bike (Hogan Health interactive system and Sony Playstation3 that could be linked to a TV monitor) and 5 games. Child-led.	13 weeks, 3 days a week, 30 minutes a day, at 60–75% of heart rate reserve	Self-reported logs of duration and frequency of use. Mean AVG play of 74.4 min^.^wk^-1^ (week 1, ~82% compliance), 19.7 min^.^wk^-1^ (weeks 3–9, ~22% compliance), and 7.8 min^.^wk^-1^ (weeks 10–13, ~9% compliance).
**AVGs plus other component interventions**			
Errickson *et al*. [41]	Provided with PlayStation 2 console, dance AVG and 2 padded dance mats. Initial in-home coaching to demonstrate the game. Series of individual coaching sessions for intervention sub-group. Families received ongoing technical support from staff. Children were provided with a camera to photograph high game scores	10 weeks. Prescribed 120 minutes of game play a week over at least 4 days. No restrictions around additional game play. 4 x 45 min coaching sessions for sub-group	Console log of number of songs and the highest grade for each song—two-thirds of memory cards were returned. Self-report log of total minutes. % completion of self-report log = 67% and 72% at week 1 and week 10 for IG1, and 82% and 41% at week 1 and week 10 for IG2. Mean weekly range of AVG use = 64 to 149 min^.^wk^-1^ (IG1) and 47 to 184 min^.^wk^-1^ (IG2). Mean AVG use for both groups declined from 164 min^.^wk^-1^ (week 1) to 64 min^.^wk^-1^(week 10). AVG use greater for IG2 vs. IG1 during first 5 weeks only (*P*<0.001).
Maloney *et al*. [42]	Provided with PlayStation 2 console, dance AVG and 2 padded dance mats (child-led). Series of 1:1 coaching sessions for intervention sub-group (coach-led) Provided with handout about operation of the game and tips for improving skills. Children were provided with stickers and a camera to log progress. Staff were contactable for technical support.	10 weeks. Prescribed 120 minutes of game play a week over at least 4 days. No restrictions around additional game play. 5 x 30 min coaching sessions for sub-group.	Self-report log of total minutes played. Console logged number of songs. Mean AVG play of 89 ± 82 min^.^wk^-1^ (~74% compliance).
Mitchell *et al*. [43, 44]	Web-based game-like physical therapy (Mitii) delivered in the home using a computer with webcam. Physical activity games interspersed with upper-limb and visual-perceptual games. Included sequences of repetitive functional exercises (e.g. alternate lunging, squatting). Health-professional-led Therapists provided ad-hoc technical support and encouragement via telephone.	20 weeks. Received 30 minutes daily for 6 days a week. Intensity based on baseline measurements and level of physical difficulty of games adjusted remotely by therapists, based on performance and feedback from participants and parents.	The number of participants regularly logging in steadily declined throughout the program, with 23% of participants logging in at the end of the intervention. Treatment dose automatically recorded by the program and monitored by therapists. Mean compliance of 54% of the potential dose.
Rubin *et al*. [45]	Parent-led curriculum of 96 preplanned PA sessions including playground games and AVGs. Provided with Wii Fit Plus and Just Dance 2 and 3 AVGs, and physical activity equipment (e.g. balls, hoops, hurdles, and cones). Regular telephone support for parents.	24 weeks. 4 days a week. Aiming to progressively achieve 25–45+ minutes of PA a day.	Parent completed checklists for playground activities—Parent and child rated the level of enjoyment and difficulty of activities completed and total duration of the session. Overall intervention compliance was 86.7%.
Staiano *et al*. [47]	Provision of Kinect and Xbox 360 console with four AVGs, a step tracker (Fitbit Zip) and a standardized curriculum booklet (child-led). Regular telehealth (video) sessions with fitness coaches (coach-led)	3 x AVGs sessions per week for 24 weeks. Curriculum guided increases in intensity and duration up to 60 mins per session.	Participants were supported by parents to record exergame play start and stop time for each challenge in the booklet. Compliance with prescribed duration—94.4% Compliance with prescribed frequency—88.5%. Steps/day recorded via a Fitbit Zip and reviewed by a fitness coach. Compliance to telehealth sessions was 92.7%.
**Non-AVG interventions**			
Tuominen *et al*. [48, 49]	Movement to music video (focused on improving motor coordination, muscle strength and aerobic fitness) via DVD or YouTube. Parent- and child-led.	8 weeks, instructed to complete videos every other day for 30 minutes	Parent completed diaries in week 1 and final week. Data not provided.

AVG = active video game

AVG = active video game; IG1 = intervention group 1; IG2 = intervention group 2.

AVG = active video game

#### Participants

Studies included a total of 1,182 participants, with the intervention group sample ranging from 10 [23] to 160 [35] participants. Mean age of participants ranged from 5 to 12 years. Most studies recruited an equal gender sample, except 2 studies where the proportion of females was <35% [35, 36].

Seven studies reported participant ethnicity, with 3 predominantly using a non-white sample—57% African American [47]; 41% Hispanic [45]; and 41% African American [37]. Three studies targeted children at risk or already classed as overweight or obese [35, 37, 47], 3 targeted children with a disability including cerebral palsy [43], a developmental coordination disorder [38], and children with non-syndromal obesity or Prader-Willi syndrome [45]. One targeted children who were insufficiently active [40].

Study design: 11 studies included a control, of which 10 were randomized, and 1 used a cross over design. Two studies compared to alternative interventions. Only 1 study measured longer-term follow-up outcomes after 14 weeks [42].

Intervention setting and mode: all interventions were delivered in the home, with 1 via the web [43]. Most interventions included AVGs (n = 12), and 1 used movement to music [48]. The AVG interventions included step-powered [36] or bike-powered [24, 40] standard video games; movement sensor input from a hand held device and camera sensor [23, 35, 37, 38, 45, 47] or a Dance Mat [23, 35, 41, 42]; or web-based video games which guided physical activity [43]. Game types included dance [23, 35, 37, 38, 41, 42, 45, 47], fitness/aerobics [35, 37, 38, 45, 47], sport [35, 37, 38, 47], kart/driving [24, 40], and immersive adventure [35, 47]. Five interventions (Table 2) included an additional intervention component of either in-person or telehealth/coaching [41–43, 47] or non-video-based/playground games using physical activity equipment provided [45]. Telehealth/coaching included general instruction and support [43, 46, 49] and support formulating solutions to barriers for physical activity [47]. The total period of intervention delivery ranged from 6 [24] to 24 [35, 45, 47] weeks, with 9 ≥12 weeks (Table 2). Seven interventions prescribed a set weekly duration of activity, ranging from 120 to 420 minutes [35, 38, 40–43, 48]. Two interventions prescribed a weekly activity duration that increased over time up to 180 minutes [47] and 315 minutes [45]. Two interventions asked participants to substitute inactive periods or inactive video game play with AVG [23, 36]. One intervention provided no guidance for frequency or duration of AVG game play [37].

Within nearly all studies, both parents and children, or children themselves, implemented the intervention. Five interventions were parent and child led, as both received intervention instructions [23, 36, 41, 45, 48], and 7 child-led, with the child receiving instructions [24, 35, 37, 38, 40, 42, 47]. The web-based AVG intervention appeared to be implemented by health professionals (physiotherapist, occupational therapist, and neuropsychologist) who remotely adjusted the level of difficulty [43].

Outcome measures: Most studies used accelerometry (n = 11) to measure physical activity behavior, over a period of 4 (n = 2) or at least 7 (n = 9) days. Of those, 10 reported MVPA and/or a range of physical activity intensities from ‘light’ to ‘vigorous’ and 1 reported counts per minute and time in physical activity at various predefined thresholds (Table 1). Two studies used self-reported duration and frequency of stationary bike use [24, 40].

### Engagement and compliance

Engagement and compliance data are presented in Table 2. All studies recorded engagement with the intervention, which was self-reported (n = 9), logged by the games console (n = 1), or a combination of both (n = 3).

One study reported significantly greater AVG engagement when supported by coaching, compared to AVGs alone [41]. Two studies reported a significant increase in AVG engagement between baseline and mid [36] or end [35] of the intervention period. Average duration of AVG play across the intervention period (where data were provided) ranged from ~90 minutes per week [42, 43] to ~280 minutes per week [23].

AVG engagement typically declined over the duration of the intervention period within several studies [35, 36, 40–43]. Where data were reported weekly, AVG engagement was greatest in the first week of the intervention [37, 40–42]. Where the earliest data collection was mid intervention, AVG engagement was greater than at the end [35, 36]. The provision of new AVGs during an intervention coincided with a ~75% increase in engagement [37] and the cessation of coaching coincided with a ~50% reduction in engagement [41]. One study observed a steady decline in the number of participants logging in to the web-based AVG over the intervention period [43].

Of the 9 studies that prescribed a frequency and/or duration of engagement with the intervention, 7 provided compliance data or engagement data (Table 2), with most achieving a mean intervention compliance of over 70% for part [40, 41] or all [38, 42, 45, 47] of the intervention duration.

Two studies highlighted issues with data quality. Errickson *et al*. [41] reported that some games console memory cards were not returned, and wide variation occurred in the completion of self-reported logs. Baranowski *et al*. [37] observed that some children in the intervention group had played inactive games, and that console data reported unlikely excessive engagement (e.g. 24 hours), could not distinguish between the study participant and other game players, and mismatched with self-reported engagement data.

### Study quality

Two studies were rated ‘weak’, 10 studies rated ‘moderate’ and 1 study rated ‘strong’. Few papers clearly stated whether or not participants were single or double blinded, resulting in a low ‘blinding’ component score for 10 studies (Table 3). Individual scores for each component are shown in Table 3.

**Table 3:** Global and component study quality assessment ratings.

	Rating of components						Global rating
Study	Selection bias	Study Design	Confounders	Blinding	Data collection methods	Withdrawals and drop-outs	
Staiano *et al*. [47]	Moderate	Strong	Strong	Moderate	Strong	Strong	**Strong**
Baranowski *et al*. [37]	Moderate	Strong	Strong	Weak	Strong	Strong	**Moderate**
Errickson *et al*. [41]	Strong	Strong	Strong	Weak	Strong	Strong	**Moderate**
Graves *et al*. [36]	Strong	Strong	Strong	Weak	Strong	Moderate	**Moderate**
Howie *et al*.[38]	Strong	Strong	Strong	Weak	Strong	Strong	**Moderate**
Maddison *et al*. [35, 39]	Moderate	Strong	Strong	Weak	Strong	Strong	**Moderate**
Maloney *et al*. [42]	Moderate	Strong	Strong	Weak	Strong	Strong	**Moderate**
Mark and Rhodes [24]	Moderate	Strong	Strong	Moderate	Weak	Strong	**Moderate**
Rhodes *et al*. [40]	Strong	Strong	Strong	Moderate	Weak	Strong	**Moderate**
Tuominen *et al*. [48, 49]	Moderate	Strong	Strong	Weak	Strong	Strong	**Moderate**
Ni Mhurchu *et al*. [23]	Moderate	Strong	Moderate	Weak	Strong	Strong	**Moderate**
Mitchell *et al*. [43, 44]	Weak	Strong	Strong	Weak	Strong	Strong	**Weak**
Rubin *et al*. [45, 46]	Strong	Weak	Strong	Weak	Strong	Strong	**Weak**
TOTAL WEAK	1	1	1	10	2	0	**2**
TOTAL MODERATE	7	0	0	3	0	1	**10**
TOTAL STRONG	5	12	12	0	11	12	**1**

### Effectiveness

The only non-AVG study included (Table 1) used home-based parent-led movement to music and was not effective [48]. Of the twelve studies incorporating AVGs, 3 showed a significant intervention effect for physical activity—1 for MVPA [47], and 2 for volume (counts per minute [23] and minutes of cycling [40]). AVGs within these included dance [23, 47] and cycling [40] activity that could be considered cardiovascular exercises. These interventions were ≥12 weeks. Two prescribed frequency, duration and intensity of game play [40, 47], while the other substituted typical video game play for AVG [23]. One study provided social support through telehealth coaching in addition to AVGs [47].

Staiano *et al*. [47] was the sole study reporting significant intervention effects post intervention (24 weeks), with a large effect size (*d* = 3.452). The significant 6-week intervention effect reported by Ni Mhurchu *et al*. [23] did not remain at 12 weeks (the end of the intervention). The significant intervention effect reported by Rhodes *et al*. [40] was only observed up to week 9 of the 13 week intervention, with the greatest group difference in week 1 (*d* = 0.52).

### Behavior change theories and techniques

Self-determination theory [50] underpinned the components of 1 intervention and social cognitive theory [51] underpinned the components of 2 interventions (Table 4). No other interventions described a theoretical underpinning. Table 4 highlights the rationale provided, and how the theory was incorporated.

**Table 4:** Behavior change theories described within interventions.

Study citation	Behavior change theory referenced and rationale provided	Incorporation into intervention
Baranowski *et al*. [37]	Self-determination theory—providing choice may enhance intrinsic motivation for behavior	The intervention allowed children to choose 1 game from a selection of 5, choose when, where and how to play the video games, and permitted them to purchase/use other video games.
Rubin *et al*. [45]	Social cognitive theory—parents may serve as a proxy and aid in the management and regulation of their child’s behavior by scheduling and facilitating opportunities for PA	The intervention was parent-led, and supported by parent and child together completing check-lists to monitor participation, and rate enjoyment and difficulty, as well as regular telephone support to aid planning and overcome barriers to implementation
Staiano *et al*. [47]	Social cognitive theory—link between behaviors (exergame play), the environment (parental and coach support) and psychosocial variables (self-efficacy and quality of life).	The intervention included social support provided by encouraging AVG play with friends and family, and promotion of self-efficacy through supportive words on the screen within the game, and within telehealth counselling sessions.

The number of Behavior Change Techniques (BCTs) identified ranged from 1 [23, 37] to 15 [45], with a mean of 6 BCTs (Table 5). Among the 3 effective studies, there were 1 [23], 8 [40] and 13 [47] BCTs identified within the intervention groups.

**Table 5:** Behavior change techniques used within intervention groups across all studies.

	Study citation													Total across studies
	Baranowski *et al*. [37]	Errickson *et al*. [41]	Graves *et al*. [36]	Howie *et al*. [38]	Maddison *et al*. [35, 39]	Maloney *et al*. [42]	Mark and Rhodes [24]	Mitchell *et al*. [43, 44]	Ni Mhurchu *et al*. [23]	Rhodes *et al*. [40]	Rubin *et al*. [45, 46]	Staiano *et al*. [47]	Tuominen *et al*. [48, 49]	
Number of BCTs	1	13	3	4	2	6	2	3	1	8	15	13	2	73
BCT and code from BCTTv1														
1.1 Goal setting (behavior)										Both groups	Parents			5
1.2 Problem solving											Parents			2
1.3 Goal setting (outcome)										Both groups				2
1.5 Review behavior goal(s)														1
2.2 Feedback on behavior														5
2.3 Self-monitoring of behavior		IG2									Children and parents			5
2.4 Self-monitoring of outcome(s) of behavior														1
2.5 Monitoring of outcome(s) of behavior without feedback														1
2.6 Biofeedback														1
3.1 Social support (unspecified)						Part of IG only								2
3.2 Social support (practical)											Parents			6
3.3 Social support (emotional)											Parents			3
4.1 Instruction on how to perform the behavior											Children and parents			4
5.4 Monitoring of emotional consequences														1
6.1 Demonstration of the behavior														1
6.3 Information about others’ approval														1
8.1 Behavioral practice/rehearsal														1
8.2 Behavioral substitution														3
8.7 Graded tasks														4
9.1 Credible source		IG2 only												2
10.0 Non-specific reward										Exergame only				2
10.2 Material reward (behavior)														1
10.4 Social reward														3
10.5 Social incentive														2
12.5 Adding objects to the environment							Both groups							12
13.1 Identification of self as role model		IG2 only									Parents			2

IG1 = intervention group 1; IG2 = intervention group. Orange = present in all probability; Green = present beyond all reasonable doubt

IG1 = intervention group 1; IG2 = intervention group. Orange = present in all probability; Green = present beyond all reasonable doubt

IG1 = intervention group 1; IG2 = intervention group. Orange = present in all probability; Green = present beyond all reasonable doubt

Twenty-six out of 93 BCTs (28%) were identified (Table 5). The coded text for BCTs is in S1 Table. Adding objects to the environment was the only BCT common across all 3 effective interventions, with goal setting (behavior) and feedback on behavior, common within Rhodes *et al*. [40] and Staiano *et al*. [47] Among all interventions, adding objects to the environment—games consoles, peripherals, physical activity equipment and music DVDs—was the most prominent (12/13 studies). Practical social support was identified across nearly half (6/13) studies, usually as technical support and coaching to facilitate implementation and provide motivation to children and/or parents. Self-monitoring of behavior was identified in over a third (5/13) of studies through regular completion of logs/diaries/checklists to monitor engagement. Feedback on behavior was also identified in over a third (5/13) of studies, which ranged from grading on accuracy [41] to information on physical activity behavior [24, 40, 47]. Goal setting (behavior) was also used in over a third (5/13) of studies, through setting a goal or encouraging a set frequency and intensity of engagement. Instructions on how to perform the behavior and graded tasks were identified in a third (4/13). BCTs were also identified within the training of parents delivering an intervention for their child [45], in addition to the BCTs identified within the intervention protocol for the child participants. BCTs were also identified within alternative intervention group protocols, for example providing additional intervention elements [41], or adding non AVG objects to the environment for control groups [24, 40].

### Process evaluations—Barriers and facilitators to engagement

Seven studies included a process evaluation, which included satisfaction surveys, [41, 42] acceptability surveys [47], interviews [37, 40, 52], and focus groups [24, 42] with children and/or parents. Children lacked motivation (reported by 32% of parents) [52], due to progressive difficulty of the intervention [52] or the AVGs being too challenging, causing frustration [24, 37, 41]. AVGs lacked sufficient variety of game choice [24], were unappealing [40], and some children found it difficult having no-one to play with [37]. Some children experienced issues with comfort of exergame bikes [24, 40] or problems with gaming equipment [40]. Participants stated competing priorities, either for the parent delivering the intervention (reported by 55% of parents) [52] or taking part in the intervention [24], or for the child directly [40, 47].

Many participants found AVGs fun and enjoyable [24, 40, 42, 47], distracted them from the physical work performed [24] and were easy to play [42, 47]. Participants reported monitoring success through using stickers and photographing high scores achieved, and had increased their game scores [42]. Having a schedule made the intervention easy to follow, and gave structure and routine [52]. Social support encouraging children to play AVGs with others led to 72% of children reporting to do so [47]. Social support from research staff gave parents a sense of belonging to the program and more parents valued this than support from other people in the home (32% vs 17% respectively) [52]. Self-efficacy was demonstrated through children’s self-belief that they were good at playing the AVGs, better than others, and satisfied with their performance [47]. Children valued having the opportunity to be more active and potentially improve their health/fitness [24, 40]. AVGs provided the opportunity for children to try activities they wouldn’t normally get to do (e.g. boxing or bowling), and meant that they “didn’t have to play outside” [37].

## Discussion

This is the first systematic review to map behavior change techniques across children’s home-based physical activity interventions. The review included 7 additional studies in comparison to previous reviews [21, 22], including 2 effective interventions, providing a timely update to the evidence base. Similar to previous reviews, there was a dominance of AVG-based interventions (12 out of 13 studies), however the more recent studies typically included additional intervention components such as telehealth support/coaching and/or physical activity equipment (Table 2). Of the 13 studies, 3 demonstrated effectiveness, but only 1 demonstrated long-term effectiveness throughout the duration of the intervention. Coding of BCTs identified the active ingredients in interventions, the most common including making physical changes to the environment, self-monitoring of behavior and practical social support. While AVGs were commonly used, the underlying intervention strategies were heterogeneous, making it difficult to identify successful characteristics. Only 1 study was rated as strong, suggesting a need for improved study design for future investigations of home-based physical activity interventions. The study aim was achieved to an extent, however, limited effectiveness and heterogeneity of studies compromised the ability to clearly identify mechanisms of action, and recommendations are focused on improving the design and evaluation of future interventions.

### Active video games as the dominant intervention mode

In line with previous reviews, there was little variation in mode of delivery, with AVGs being the dominant mode [21, 22], While laboratory-based studies established adolescents found AVGs enjoyable, and game play elevated energy expenditure to moderate or vigorous intensity [53, 54], highly controlled efficacy studies may lack external validity. The lack of effectiveness among most interventions in this review suggests that observations during a controlled single dose of AVG play may not transfer to real world settings and sustained behavioral engagement. This is likely explained by uncontrollable factors within the physical and social environment. More recently, interventions targeting children’s physical activity at home have used a multi-component approach, supplementing AVG play with regular support from research staff or fitness coaches either in the home or via telephone. The most effective intervention in this review supported AVG play with regular coach-led telehealth sessions [47].

### Mechanisms of effectiveness

The limited number of effective studies, wide variation in study design and intervention used, and dominance of low to moderate quality studies made it difficult to reach consensus on mechanisms of effectiveness. AVGs were considered fun and enjoyable [24, 40, 42, 47], suggesting that game play provided intrinsic motivation. However initial high engagement in AVGs was typically not sustained for the duration of interventions [23, 36, 37, 40–42], suggesting that additional mechanisms are important to overcome the novelty factor and support sustained behavior change. Focusing on the 1 high quality study [47], retention and compliance were high, and physical activity behavior change was sustained throughout the intervention. The focus on building social support through telehealth coaching and playing AVGs with others may have been important components contributing to the effectiveness. Social support through interaction with others may be important for sustaining children’s motivation for AVG, considering the greater dropout observed with single player vs. multiplayer AVGs games [55]. Social support from activity mentors and other participants joining home-based online physical activity sessions was reported as facilitators for engaging adolescent girls [56].

The intervention used by Staiano *et al*. [47] was underpinned by the social cognitive theory, including a focus on building self-efficacy within coaching and the AVG, and the study reported a significant increase in self-efficacy alongside increased physical activity. Self-efficacy has been highlighted as an important mechanism within children’s physical activity interventions [57, 58], and important for good AVG design [59]. The difficulties reported by children in relation to AVG play within the non-effective interventions [24, 37, 41, 52] may have led to low self-efficacy, limiting the effectiveness of the intervention.

Although the mechanisms are unclear, the use of regular video game players may have contributed to the effectiveness reported by other studies in the review [23, 40] given that a similar population showed sustained AVG engagement over a 24 week intervention [35].

### Behavior change techniques

Within the 3 effective interventions, the most effective used the greatest number of BCTs [47]. However the highest number of BCTs across all studies was coded within a non-effective intervention [45]. This suggests that the type of BCTs, and/or implementation of BCTs, may be more important than the number used. Although 9 BCTs were common between these two interventions, many of these were coded within the telehealth component of the intervention which was implemented with parents in the non-effective study, suggesting that active involvement of children in telehealth coaching may be important to sustain engagement and enhance effectiveness.

Among the most common BCTs identified through this review, the only BCTs applied with some consistency across interventions (S3 Table) were ‘adding objects to the environment’, ‘self-monitoring of behavior’, and ‘goal setting (behavior)’. Other common BCTs showed wide variation in their implementation (S3 Table), for example, the extent of practical social support ranged from external technical support [38, 43], to at-home visits to setup gaming equipment [42, 47] and requiring children play AVGs with others [47]. The inconsistent implementation of BCTs limits the ability to identify active ingredients for effectiveness.

### Enhancing intervention quality, acceptability and effectiveness

All 3 effective AVG interventions included lower body movements (dance or cycling), eliciting greater energy expenditure than AVGs involving primarily upper body movements [60]. AVGs involving lower body movements may maximize the potential of increasing MVPA, and should be tested within rigorously designed studies. Further research is also needed to understand how motivation for playing AVGs and enhancing PA can be sustained over time. Recent research on the use of animated narrative AVGs to enhance children’s MVPA [61] and the identification of principles for best practice in AVG design should inform choice of AVG in future interventions [62].

The use of social support and building self-efficacy using AVGs and telehealth coaching may be important mechanisms for effectiveness, and should be explored further. However, this may not be affordable or scalable to population level, suggesting other modes of intervention should be considered. In a multi-setting intervention with adolescent females completing live online workouts at home, other participants and scheduled messages from researchers provided social support, and motivated participants to complete physical activity sessions [56].

Only 28% of the 93 possible BCTs were identified within the 13 studies, highlighting the opportunity to develop novel interventions that incorporate other BCTs that may enhance effectiveness. For example, previously identified barriers of parental concerns around injury or damage to the home [63] and competing priorities [24, 40, 47, 52] could be addressed by restructuring the environment (BCT 12.1) and BCTs influencing reflective motivation such as providing information about health consequences (BCT ‘5.1), and feedback on the outcomes of behavior (BCT 2.7) [26].

Only 2 studies report using insight from children and parents to influence the design of the intervention [39, 46]. Co-producing interventions may enhance the feasibility, acceptability, quality, and impact of these interventions [64].

### Study design and reporting of outcomes

Of the 13 studies included, 12 (92%) were rated low-moderate quality, with blinding of assessors and participants either not described, or not implemented (85%), and potential selection bias in the recruitment process (62%). Improving study design could reduce variability in study outcomes and help strengthen the evidence base. Few studies reported effect sizes, descriptive outcomes data and ethnicity of participants (Table 1). The use of the Consolidated Standards of Reporting Trials [65] would support methodological rigor and ensure outcome data is sufficiently reported, which may facilitate understanding effectiveness across different ethnic groups.

Most interventions measured physical activity using accelerometry, which will likely have included physical activity away from the home. Combining Global Positioning System (GPS) unit location data with heart rate [66] and/or accelerometry data [67] and indoor location and movement sensors [68] could be explored to improve the quality of objective measurement of home-based PA. Many studies did not measure short-term effectiveness (Tables 1 and 2) [38, 41–43, 45, 47] limiting the ability to identify potential initial engagement or effectiveness [47]. Weekly reporting of engagement and effectiveness would provide further insight into short- and medium-term effectiveness. Similarly, only 1 study measured outcomes following cessation of the intervention [42]. Follow-up measures of effectiveness could further knowledge around sustainability of behavior.

Although 3 studies referred to behavior change theory, none explicitly referred to using BCTs within their intervention design, limiting understanding of how theory was implemented within the intervention. Few studies provided a detailed description of the intervention and AVG play, or supplied copies of printed/online resources. Common BCTs within AVGs such as reinforcement and guided practice which can build self-efficacy may have been missed from coding [69]. Supplying copies of intervention materials/resources and use of the Template for Intervention Description and Replication [70] checklist would aid reviewers with the coding of BCTs.

Only 7 studies (58%) explored intervention barriers and facilitators. Future interventions should use a more mixed-method approach to help understand how an intervention has been effective [71, 72], especially within complex multi-component interventions [73].

### Strengths and limitations of the study

The use of extensive search criteria and only including controlled, peer-reviewed studies ensured high-quality studies, with only two rated weak by the quality assessment tool for quantitative studies [29]. However, excluding uncontrolled studies may have omitted more varied home-based physical activity interventions. The review was also limited to English language publications and dominated by studies conducted in high income countries with majority White ethnic group populations, limiting the application of findings across low and middle income countries and among minority ethnic groups.

The review adhered to the updated PRISMA guidelines [27], which ensured transparent reporting and allows for future replication of the methods. A meta-analysis was not feasible due to heterogeneity in study designs and inconsistent reporting of effect estimates within studies.

The study also included an evaluation of BCTs used across interventions, and process evaluation data, helping synthesize intervention components which may be required for success. However, the low number of studies included in the review limits the ability to reach consensus on effective BCTs.

## Conclusion

Many children are not sufficiently active for health, and their physical activity declined during the COVID-19 pandemic when required to stay at home. This review provides a timely update and renewed synthesis of the literature on interventions targeting children’s physical activity at home. This review highlights the limited evidence base for increasing children’s physical activity at home, with the majority of studies focused on AVGs using low to moderate quality study designs, and interventions which fail to sustain engagement. There is some evidence that the addition of telehealth coaching may enhance effectiveness through social support and building self-efficacy, however further high quality studies are needed, with greater inclusion of minority ethnic groups. Future studies should also explore non-AVG interventions and implement BCTs addressing barriers identified within the existing literature. Understanding how to effectively facilitate children’s physical activity within the home could contribute towards increased MVPA, particularly for those children who spend more time at home, and where increased cost of living restricts physical activity opportunities. It may also mitigate reductions in physical activity during future pandemics through informing polices and practice.

## Supporting information

S1 Checklist KIN 4400 independent research study in kinesiology | PRISMA 2020 checklist. (DOCX)

S1 Table Search strategies used in databases and number of results obtained across each search period. (DOCX)

S2 Table Overview of studies excluded in the final stage of review. (DOCX)

S3 Table Text coded for identification of behavior change techniques. (DOCX)
