# Supplementary material for: Interventions targeting children and young people’s physical activity behavior at home: A systematic review
Source: PLoS One. 2023 Aug 9;18(8):e0289831. doi: 10.1371/journal.pone.0289831 (PMC10411747; doi:10.1371/journal.pone.0289831)
Supplement: S1 Checklist — (DOCX) [file pone.0289831.s001.docx]

| **Section and Topic** | **Item #** | **Checklist item** | **Location where item is reported** |
| --- | --- | --- | --- |
| **TITLE** | | |  |
| Title | 1 | Identify the report as a literature review. | Title; page 1. |
| **ABSTRACT** | | |  |
| Abstract | 2 | Provide a structured summary including, as applicable: background; objectives; data sources; study eligibility criteria, participants, and interventions; study appraisal and synthesis methods; results; limitations; conclusions and implications of key findings.  See the [PRISMA 2020 for Abstracts checklist](http://www.prisma-statement.org/Extensions/Abstracts.aspx) for the complete list. | Abstract; pages 2-3. |
| **INTRODUCTION** | | |  |
| Rationale | 3 | Describe the rationale for the review in the context of existing knowledge, i.e., what is already known about your topic. | Introduction pages 4-5, lines 48-97. |
| Objectives | 4 | Provide an explicit statement of the objective(s) or question(s) the review addresses with reference to participants, interventions, comparisons, outcomes, and study design (PICOS). | Introduction; pages 6, lines 98-102.  Methods - Eligibility criteria; page 6; lines 111-123. |
| **METHODS** | | |  |
| Eligibility criteria | 5 | Specify the inclusion and exclusion criteria for the review and how studies were grouped for the syntheses with study characteristics (e.g., PICOS, length of follow-up) and report characteristics (e.g., years considered, language, publication status) used as criteria for eligibility, giving rationale. | Methods - Eligibility criteria; page 6, lines 111-134.  Methods - Data synthesis; page 10, lines 196-204. |
| Information sources | 6 | Specify all databases, registers, websites, organisations, reference lists and other sources searched or consulted to identify studies. Specify the date when each source was last searched or consulted. | Methods - Search strategy; page 7, lines 137-140.  S1 Table |
| Search strategy | 7 | Present the full search strategies for all databases, registers and websites, including any filters and limits used. | S1 Table |
| Selection process | 8 | State the process for selecting studies (i.e., screening, eligibility).  Specify the methods used to decide whether a study met the inclusion criteria of the review, including how many reviewers screened each record and each report retrieved, whether they worked independently, and if applicable, details of automation tools used in the process. | Methods - Selection process; page 8, lines 154-162. |
| Study risk of bias assessment | 11 | Specify the methods used to assess risk of bias in the included studies, including details of the tool(s) used, how many reviewers assessed each study and whether they worked independently, and if applicable, details of automation tools used in the process. | Methods - Quality of studies; page 9, lines 188-193. |
| **RESULTS** | | |  |
| Study selection | 16a | Describe the results of the search and selection process, from the number of records identified in the search to the number of studies included in the review, ideally using a flow diagram. | Results; page 10, lines 207-215.  Figure 1; page 10, line 210.  S1 Table. |
|  | 16b | Cite studies that might appear to meet the inclusion criteria, but which were excluded, and explain why they were excluded. | Results; page 10, lines 213-214.  S2 Table |
| Study characteristics | 17 | Cite each included study and present its characteristics (e.g., study size, PICOS, follow-up period). | Results - Study and intervention characteristics; page 11, lines 225-229; pages 18-19, lines 256-293.  Table 1; pages 12-15.  Table 2; pages 16-18. |
| Risk of bias in studies | 18 | Present assessments of risk of bias for each included study. | Results - Study quality; page 21, lines 324-327.  Table 3; page 22 |
| Results of individual studies | 19 | For all outcomes, present, for each study: (a) summary statistics for each group (where appropriate) and (b) an effect estimate and its precision (e.g. confidence/credible interval), ideally using structured tables or plots. | Results – Effectiveness; pages 22-23, lines 332-344. N.B. Insufficient reporting of data within some studies.  Table 1; pages 12-15.  Results – Behaviour change theories and techniques; pages 23-24, lines 347-374.  Table 4; page 23.  Table 5; pages 25-27.  S3 Table. |
| **DISCUSSION** | | |  |
| Discussion | 23a | Provide a general interpretation of the results in the context of other evidence. | Discussion - Active video games as the dominant intervention mode; pages 29-30, lines 427-437.  Discussion – Mechanisms of effectiveness; pages 30-31, lines 440-464.  Discussion – Behaviour change techniques; page 31, lines 467-481.  Discussion – Enhancing intervention quality, acceptability and effectiveness; page 32, lines 484-507. |
|  | 23b | Discuss any limitations of the evidence included in the review. | Discussion - Active video games as the dominant intervention mode; pages 29-30, lines 427-437.  Discussion – Mechanisms of effectiveness; pages 30-31, lines 440-464.  Discussion – Behaviour change techniques; page 31, lines 467-481.  Discussion – Enhancing intervention quality, acceptability and effectiveness; page 32, lines 484-507.  Discussion – study design and reporting; pages 33-34, lines 540-538. |
|  | 23c | Discuss any limitations of the review processes used. | Discussion – Strengths and limitations of the study; page 34, lines 541-554. |
|  | 23d | Discuss implications of the results for practice, policy, and future research. | Discussion - Enhancing intervention quality, acceptability and effectiveness; page 32, lines 484-504.  Discussion – study design and reporting; pages 33-34, lines 540-538.  Conclusion; page 35, lines 557-570. |
| **OTHER INFORMATION** | | |  |
| Registration and protocol | 24a | Provide registration information for the review, including register name and registration number, or state that the review was not registered. | Abstract ; page 3, line 47.  Methods; page 6, line 106. |
|  | 24b | Indicate where the review protocol can be accessed, or state that a protocol was not prepared. | Methods; page 6, line 107. |
|  | 24c | Describe and explain any amendments to information provided at registration or in the protocol. | Methods; page 6, line 107. |
| Support | 25 | Describe sources of financial or non-financial support for the review, and the role of the funders or sponsors in the review. | In submission process (omitted from manuscript as required in submission guidelines) and in response to reviewer comments letter. |
| Competing interests | 26 | Declare any competing interests of review authors. | In submission process (omitted from manuscript as required in submission guidelines) |
| Availability of data, code, and other materials | 27 | Report which of the following are publicly available and where they can be found: template data collection forms; data extracted from included studies; data used for all analyses; analytic code; any other materials used in the review. | Supporting information; page 45, lines 818-821.  Files submitted separately within the submission process. |
