# Supplementary material for: Interventions targeting children and young people’s physical activity behavior at home: A systematic review
Source: PLoS One. 2023 Aug 9;18(8):e0289831. doi: 10.1371/journal.pone.0289831 (PMC10411747; doi:10.1371/journal.pone.0289831)
Supplement: S1 Table — (DOCX) [file pone.0289831.s002.docx]

**S1 Table. Search strategies used in databases and number of results obtained across each search period**

| **Database** | **Coverage** | **Search terms** | **Search 1 - date (results)** | **Search 2 - date (results)** | **Search 3 - date (results)** |
| --- | --- | --- | --- | --- | --- |
| Ovid - Embase, AMED & PsychINFO | Embase - 1974 to present; AMED - 1985 to present; PsychINFO - 1806 to present | ((Child or "young person" or adolescence or adolescents or teen or girl or boy or infant or preschool or family or toddler) and (Home or community or family or school or “primary care” or digital or app or online or web or parent or mother or father or indoor or garden or yard) and ("physical activity" or exercise or fitness or play or move or dance) and ("step count" or accelerometer or pedometer or GPS or "global positioning system" or "moderate to vigorous physical activity" or MVPA or minutes) and (Intervention* or toolkit or resource or campaign or promotion or trial or "randomised controlled trial" or "controlled trial" or RCT or "primary prevention" or strategy or program or experiment or quasi)).af. | 23/09/2020 (12,645) | 11/09/2021 (1,927) | 27/06/2022 (585) |
| Scopus | 1788 to present | ( TITLE-ABS-KEY ( ( child OR "young person" OR adolescence OR adolescents OR teen OR girl OR boy OR infant OR preschool OR family OR toddler ) ) AND TITLE-ABS-KEY ( ( home OR community OR family OR school OR primary AND care OR digital OR app OR online OR web OR parent OR mother OR father OR indoor OR garden OR yard ) ) AND TITLE-ABS-KEY ( ( "physical activity" OR exercise OR fitness OR play OR move OR dance ) ) AND TITLE-ABS-KEY ( ( "step count" OR accelerometer OR pedometer OR gps OR "global positioning system" OR "moderate to vigorous physical activity" OR mvpa OR minutes ) ) AND TITLE-ABS-KEY ( ( intervention* OR toolkit OR resource OR campaign OR promotion OR trial OR "randomised controlled trial" OR "controlled trial" OR rct OR "primary prevention" OR strategy OR program OR experiment OR quasi ) ) ) | 11/08/2020 (2,135) | 23/09/2021 (356) | 27/06/2022 (783) |
| Web of Science | 1999 to present | ALL FIELDS: ((Child OR "young person" OR adolescence OR adolescents OR teen OR girl OR boy OR infant OR preschool OR family OR toddler) ) AND ALL FIELDS: ((Home OR community OR family OR school OR primary care OR digital OR app OR online OR web OR parent OR mother OR father OR indoor OR garden OR yard) ) AND ALL FIELDS: (("physical activity" OR exercise OR fitness OR play OR move OR dance) ) AND ALL FIELDS: (("step count" OR accelerometer OR pedometer OR GPS OR "global positioning system" OR "moderate to vigorous physical activity" OR MVPA OR minutes) ) AND ALL FIELDS: ((Intervention* OR toolkit OR resource OR campaign OR promotion OR trial OR "randomised controlled trial" OR "controlled trial" OR RCT OR "primary prevention" OR strategy OR program OR experiment OR quasi) )  Refined by: LANGUAGES: ( ENGLISH )  Indexes=SCI-EXPANDED, SSCI, A&HCI, CPCI-S, CPCI-SSH, ESCI Timespan=All years | 11/08/2020 (5,950) | 11/09/2021 (839) | 27/06/2022 (531) |
| Cochrane library | 1992 to present | (Child OR "young person" OR adolescence OR adolescents OR teen OR girl OR boy OR infant OR preschool OR family OR toddler) in All Text AND (Home OR community OR family OR school OR primary care OR digital OR app OR online OR web OR parent OR mother OR father OR indoor OR garden OR yard) in All Text AND ("physical activity" OR exercise OR fitness OR play OR move OR dance) in All Text AND ("step count" OR accelerometer OR pedometer OR GPS OR "global positioning system" OR "moderate to vigorous physical activity" OR MVPA OR minutes) in All Text AND (Intervention* OR toolkit OR resource OR campaign OR promotion OR trial OR "randomised controlled trial" OR "controlled trial" OR RCT OR "primary prevention" OR strategy OR program OR experiment OR quasi) | 25/08/2020 (1,454) | 22/09/2021 (576) | 27/06/2022 (13) |

| **Database** | **Coverage** | **Search terms** | **Search 1 - date (results)** | **Search 2 - date (results)** | **Search 3 - date (results)** |
| --- | --- | --- | --- | --- | --- |
| PubMed/Medline | 1966 to present/1946 to present | ((((Child or "young person" or adolescence or adolescents or teen or girl or boy or infant or preschool or family or toddler) AND (Home or community or family or school or "primary care" or digital or app or online or web or parent or mother or father or indoor or garden or yard)) AND ("physical activity" or exercise or fitness or play or move or dance)) AND ("step count" or accelerometer or pedometer or GPS or "global positioning system" or "moderate to vigorous physical activity" or MVPA or minutes)) AND (Intervention* or toolkit or resource or campaign or promotion or trial or "randomised controlled trial" or "controlled trial" or RCT or "primary prevention" or strategy or program or experiment or quasi) | 12/08/2020 (8,743) | 23/09/2021 (190) | 27/06/2022 (311) |
| EBSCO (CINAHL & SPORTDiscus) | CINAHL -1981 to present; SPORTDiscus - 1800 to present | TX ( (Child OR "young person" OR adolescence OR adolescents OR teen OR girl OR boy OR infant OR preschool OR family OR toddler) ) AND TX ( (Home OR community OR family OR school OR primary care OR digital OR app OR online OR web OR parent OR mother OR father OR indoor OR garden OR yard) ) AND TX ( ("physical activity" OR exercise OR fitness OR play OR move OR dance) ) AND TX ( ("step count" OR accelerometer OR pedometer OR GPS OR "global positioning system" OR "moderate to vigorous physical activity" OR MVPA OR minutes) ) AND ( (Intervention* OR toolkit OR resource OR campaign OR promotion OR trial OR "randomised controlled trial" OR "controlled trial" OR RCT OR "primary prevention" OR strategy OR program OR experiment OR quasi) ) | 22/09/2020 (6,550) | 22/09/2021 (401) | 27/06/2022 (587) |
| **TOTAL** | | | 34,477 | 4,289 | 2,810 |
