# Supplementary material for: Interventions targeting children and young people’s physical activity behavior at home: A systematic review
Source: PLoS One. 2023 Aug 9;18(8):e0289831. doi: 10.1371/journal.pone.0289831 (PMC10411747; doi:10.1371/journal.pone.0289831)
Supplement: S2 Table — (DOCX) [file pone.0289831.s003.docx]

**S2 Table. Overview of studies excluded in the final stage of review**

| **Study** | **Participants** | **Study design. Intervention setting and mode** | **Physical activity outcome measure** | **Exclusion reason** |
| --- | --- | --- | --- | --- |
| Duncan *et al.*^30^ | 675 children (aged 7-10 years) | RCT. 8-week (diet and physical activity intervention). Curriculum-based homework schedule and in-class teaching resource. | Pedometer (pre, post and 6-month follow-up) - separate pedometers used for school hours and outside school hours | Home-based component included the promotion of activity away from the home (e.g. swimming, family walks in the neighbourhood). Pedometer data cannot be used to identify change in PA at home |
| Okely *et al*.^31^ | 658 children (mean age 3.8 years) | RCT. 18-month childcare- and home-based. Physical education, activity learning, dance (delivered by educators, but parents participated in the same activities at home with children). | Accelerometer (baseline and 6 months)  Parent-completed checklists to reflect engagement with home-based component | Insufficient completion of checklists. Baseline attempts using accelerometers to measure home-based PA proved too challenging to repeat post-intervention. |
| Pearce and Dollman^32^ | 147 children (aged 8-13 years) | Control trial (not randomised). 10 x 1h school-based learning and physical activity, home-based activity programme, and parent lifestyle workshop. | Accelerometer (Baseline, post and 10-week follow-up)  Physical Activity Questionnaire-Children | Not possible to separate accelerometer PA outcomes at school and home. PAQ-C does not include measures of physical activity at home.  The home-based component was not sufficiently described and suggests the inclusion of activities away from the home (unable to receive a response from the author to confirm this). |
| Smith, Petosa and Sexton^33^ | 52 children (mean age 12.8 years) | Pilot study. 10-week school-based mentoring programme to learn and practice skills they could do in their bedrooms or homes without equipment | Accelerometer (pre and post) - worn outside of school hours only | Lack of comparator group |
| Tuominen *et al*.^34^ | 15 families (4-6 year olds and their parents | Pilot study. 8-week home-based exercise on a music mat | Accelerometer (baseline, week 1, post)  Exercise diaries. | Lack of comparator group |
