# Supplementary material for: Interventions targeting children and young people’s physical activity behavior at home: A systematic review
Source: PLoS One. 2023 Aug 9;18(8):e0289831. doi: 10.1371/journal.pone.0289831 (PMC10411747; doi:10.1371/journal.pone.0289831)
Supplement: S3 Table — (DOCX) [file pone.0289831.s004.docx]

**S3 Table. Text coded for identification of Behavior Change Techniques^*^**

**^*^Behavior Change Techniques (BCTs) were implemented within intervention groups only unless specified**

| **BCT** | **Study** | **Text coded** |
| --- | --- | --- |
| 1.1 Goal setting (behaviour) | Howie *et al.*^38^ | +, article (Children were encouraged to play the AVGs for at least 20 min a day on most days of the week (i.e. 4–5 days) |
|  | Maloney *et al.*^42^ | +. Article (a 120mpw goal was chosen) |
|  | Rhodes *et al.*^40^ | Intervention group (exergame); ++. Article (The recommended exercise training…. 3 day/week for 30 min/day) |
|  |  | Control (stationary bike); ++. Article (The recommended exercise training…. 3 day/week for 30 min/day) |
|  | Rubin *et al.*^45,46^ | Parent training; ++, article (...and goal setting are incorporated into the baseline training of parents and during the follow-up phone calls) |
|  | Staiano *et al.*^47^ | ++, article (The GameSquad intervention encouraged participants to meet a goal of 60 minutes/day of MVPA for 24-weeks) |
| 1.2 Problem solving | Rubin *et al.* ^45,46^ | Parent training;- ++, article (along with discussions about planning and overcoming barriers into the parent training component of the program) |
|  | Staiano *et al.*^47^ | ++, article (helped the child and parent to create solutions to barriers for physical activity.) |
| 1.3 Goal setting (outcome) | Rhodes *et al.*^40^ | Intervention group (exergame); ++, article (The recommended exercise training regime for ….60–75% of heart rate reserve |
|  |  | Control (stationary bike); ++, article (The recommended exercise training regime for ….60–75% of heart rate reserve |
|  | Rubin *et al.* ^45,46^ | +, article (no. of mins play per week = from 25 to 45 mins) |
| 1.5 Review behaviour goal(s) | Staiano *et al.*^47^ | ++, article (The fitness coach followed a script for the virtual meetings that reviewed child’s steps/day) |

+ = present in all probability; ++ = present beyond all reasonable doubt. IG1 =intervention group 1; IG2 = intervention group

| **BCT** | **Study** | **Text coded** |
| --- | --- | --- |
| 2.2 Feedback on behaviour | Errickson *et al.*^41^ | IG1; ++, article (being graded on accuracy) |
|  |  | IG2; ++, article (being graded on accuracy) |
|  | Mark & Rhodes^24^ | +, article (stationary bicycle that reads both speed and steering) |
|  | Rhodes *et al.*^40^ | +, The Hoggan Health interactive video gaming system reads The participant’s speed (measured by cycling cadence) and steering |
|  | Rubin *et al.* ^45,46^  Staiano *et al.*^47^ | +, article (during which feedback was offered to families to sustain motivation for participation) |
|  |  | ++, Fitbit zip (provided a Fitbit Zip (Fitbit, San Francisco, California)) |
|  | Staiano *et al.*^47^ | ++, article (In this booklet, children with parental assistance recorded exergame play start and stop time for each challenge) |
| 2.4 Self-monitoring of outcome(s) of behaviour | Rhodes *et al.*^40^ | Intervention group (exergame); ++, article (Participants were provided written and verbal instructions on the ratings of perceived exertion (RPE) associated with the recommended training intensity and received heart rate monitors to support participant fidelity to the target intensity) |
|  |  | Control (stationary bike); ++, article (Participants were provided written and verbal instructions on the ratings of perceived exertion (RPE) associated with the recommended training intensity and received heart rate monitors to support participant fidelity to the target intensity) |
| 2.5 Monitoring of outcome(s) of behaviour without feedback | Tuominen *et al.*^48,49^ | Intervention; +, article (accelerometer) |
|  |  | Control; +, article (accelerometer) |
| 2.6 Biofeedback | Rhodes *et al.*^40^ | Intervention group (exergame); ++, article (Participants were provided written and verbal instructions on the ratings of perceived exertion (RPE) associated with the recommended training intensity and received heart rate monitors to support participant fidelity to the target intensity) |
|  |  | Control (stationary bike); ++, article (Participants were provided written and verbal instructions on the ratings of perceived exertion (RPE) associated with the recommended training intensity and received heart rate monitors to support participant fidelity to the target intensity) |

+ = present in all probability; ++ = present beyond all reasonable doubt. IG1 =intervention group 1; IG2 = intervention group

| **BCT** | **Study** | **Text coded** |
| --- | --- | --- |
| 3.1 Social support (unspecified) | Maloney *et al.*^42^ | +, article (weekly 1:1 coaching sessions) - HALF OF SAMPLE ONLY |
|  | Rubin *et al.* ^45,46^ | ++, article (Further, self-efficacy in the children will be targeted through parents serving as skilled or learning models for the activity and providing verbal encouragement throughout the program) |
| 3.2 Social support (practical) | Errickson *et al.*^41^ | IG1; ++, article (coach helped the child understand the letter grade assigned and the number of ‘‘perfects’’ and ‘‘greats’’ received) |
|  |  | IG2; ++, article (coach helped the child understand the letter grade assigned and the number of ‘‘perfects’’ and ‘‘greats’’ received) |
|  | Howie *et al.*^38^ | +, article (Research assistants contacted participants every 2 weeks during both periods to troubleshoot technical issues) |
|  | Maloney *et al.*^42^ | ++, article (on 8-MB Playstation2 memory cards which we collected at week 10. Staff set up the equipment in the home, ensured it was operational, and provided the child and caregivers with a brief handout about operation of the game and strategies for improving skills. Research team members were available by pager to address equipment malfunctions) |
|  | Mitchell *et al.*^43,44^ | ++, article (The level of difficulty was adjusted remotely by therapists (physiotherapist, occupational therapist, and neuropsychologist) who were available to the participants and their families via e-mail, telephone, or video-conferencing to provide encouragement and technical support) |
|  | Rubin *et al.* ^45,46^ | Parent training; ++, article (Over the 24 wk, parents received telephone calls from a member of the research team that followed a common script; PA counselling and troubleshooting related to curriculum implementation and motivation was provided during these telephone calls) |
|  | Staiano *et al.*^47^ | ++, article (The GameSquad intervention provided social support by requiring children to play with or against a family member or friend, Two fitness coaches visited the parent and child at home within 7- days of randomization to deliver and set up the gaming equipment and play the first gaming challenge together) |
| 3.3 Social support (emotional) | Mitchell *et al.*^43,44^ | ++, article (The level of difficulty was adjusted remotely by therapists (physiotherapist, occupational therapist, and neuropsychologist) who were available to the participants and their families via e-mail, telephone, or video-conferencing to provide encouragement and technical support) |
|  | Rubin *et al.* ^45,46^ | Parent training; ++, article (Over the 24 wk, parents received telephone calls from a member of the research team that followed a common script; PA counselling and troubleshooting related to curriculum implementation and motivation was provided during these telephone calls) |
|  | Staiano *et al.*^47^ | ++, article (by requiring children and parents to attend telehealth counselling sessions) |

+ = present in all probability; ++ = present beyond all reasonable doubt. IG1 =intervention group 1; IG2 = intervention group

| **BCT** | **Study** | **Text coded** |
| --- | --- | --- |
| 4.1 Instruction on how to perform the behaviour | Errickson *et al.*^41^ | IG1; ++, article (the coach explained the concept of DDR with a familiar metaphorical concept of…" |
|  |  | IG2; ++, article (the coach explained the concept of DDR with a familiar metaphorical concept of…" |
|  | Maloney *et al.*^42^ | ++, article (brief handout about operations of the game) |
|  | Rhodes *et al.*^40^ | Intervention group (exergame); ++, article (Participants were provided written and verbal instructions on the ratings of perceived exertion (RPE) associated with the recommended training intensity and received heart rate monitors to support participant fidelity to the target intensity) |
|  |  | Control (stationary bike); ++, article (Participants were provided written and verbal instructions on the ratings of perceived exertion (RPE) associated with the recommended training intensity and received heart rate monitors to support participant fidelity to the target intensity) |
|  | Rubin *et al.* ^45,46^ | Intervention; ++, article (participants were trained on the use of the curriculum during the first visit) |
|  |  | Parent training; ++, article (parents will be trained on the delivery of the Active Play @ Home intervention, instructional cues and modifications to assist the parent in leading the activities for his/her child) |

| 5.4 Monitoring of emotional consequences | Rubin *et al.* ^45,46^ | ++, article (Parents and children were asked to rate the level of enjoyment and difficulty of activities completed and to indicate the total duration of the session.) |
| --- | --- | --- |
| 6.1 Demonstration of the behaviour | Errickson *et al.*^41^ | IG1; ++, article (coach demonstrated each action) |
|  |  | IG2; ++, article (coach demonstrated each action) |

| 6.3 Information about others' approval | Errickson *et al.*^41^ | IG1; ++, article (graded on accuracy) |
| --- | --- | --- |
|  |  | IG2; ++, article (graded on accuracy) |

| 8.1 Behavioural practice/rehearsal | Errickson *et al.*^41^ | IG1; ++, article (had the child practise the action) |
| --- | --- | --- |
|  |  | IG2; ++, article (had the child practise the action) |

+ = present in all probability; ++ = present beyond all reasonable doubt. IG1 =intervention group 1; IG2 = intervention group

| **BCT** | **Study** | **Text coded** |
| --- | --- | --- |
| 8.2 Behavioural substitution | Graves *et al.*^36^ | +, article ( peripheral device (jOG) encourages step-powered gaming on multiple games) |
|  | Maddison *et al.*^35,39^ | ++, article (substituting periods of traditional non-active video game play with the active version) |
|  | Staiano *et al.*^47^ | +, article (Exergames (i.e. video games that require physical activity) transform sedentary screen-time into physically active screen-time.) |
| 8.7 Graded tasks | Errickson *et al.*^41^ | IG1; ++, article (Coaches went to the next lesson after youth mastered that level, ensuring that a certain amount of motor learning has been attained before moving to a more complex level) |
|  |  | IG2; ++, article (Coaches went to the next lesson after youth mastered that level, ensuring that a certain amount of motor learning has been attained before moving to a more complex level) |
|  | Mitchell *et al.*^43,44^ | ++. Article (These were incremented weekly by the physiotherapist by increasing the repetitions, speed, step height, and balance challenge in response to individual performance and feedback from the participants and their parents or caregivers.) |
|  | Rubin *et al.* ^45,46^ | ++, article (the duration of PA sessions was systematically progressed from 25 to 45+ min of MVPA over the course of the 24 wk) |
|  | Staiano *et al.*^47^ | ++, article (Each exergaming participant received a booklet that provided a standardised gameplay curriculum to play three challenges each week with increasing intensity, difficulty, and duration (10 min/session in week 1, increasing by 10 min each session and sustained at 60 min/session after week 6) |
| 9.1 Credible source | Errickson *et al.*^41^ | IG2; +, article (involvement of the coach) |
|  | Staiano *et al.*^47^ | ++, article (meeting with fitness coach for the first 6 weeks) |
| 10.0 Non-specific reward | Graves *et al.*^36^ | +, article (For every step the pedometer records, 1-s of onscreen movement is obtained, For continuous gaming, sustained stepping is required) |
|  | Rhodes *et al.*^40^ | +, article (Thus participants move the avatar of the game through pedalling and steer the avatar with handlebars on the cycle ergometer.) |

+ = present in all probability; ++ = present beyond all reasonable doubt. IG1 =intervention group 1; IG2 = intervention group

| **BCT** | **Study** | **Text coded** |
| --- | --- | --- |
| 10.2 Material reward (behaviour) | Rubin *et al.* ^45,46^ | ++, article (youth received a $60 gift card to a department store if they completed 70% of the PA sessions) |
| 10.4 Social reward | Errickson *et al.*^41^ | IG1; ++, article (the number of ‘‘perfects’’ and ‘‘greats’’ received) |
|  |  | IG2; ++, article (the number of ‘‘perfects’’ and ‘‘greats’’ received) |
|  | Rubin *et al.* ^45,46^ | ++, article (Further, self-efficacy in the children will be targeted through parents serving as skilled or learning models for the activity and providing verbal encouragement throughout the program) |
|  | Staiano *et al.*^47^ | ++, article (Exergames encourage exercise through supportive words on the screen like “Flawless") |
| 10.5 Social incentive | Errickson *et al.*^41^ | IG1; +, article (the number of ‘‘perfects’’ and ‘‘greats’’ received) - they can anticipate this thus seen as an incentive |
|  |  | IG2; +, article (the number of ‘‘perfects’’ and ‘‘greats’’ received)- they can anticipate this thus seen as an incentive |
|  | Staiano *et al.*^47^ | +, article (Exergames encourage exercise through supportive words on the screen like “Flawless") |
| 12.5 Adding objects to the environment | Baranowski *et al.*^37^ | ++, article (each participating child was provided with a Wii console and an active video game) |
|  | Errickson *et al.*^41^ | IG1; ++, article (unlimited access to the game) |
|  |  | IG2; ++, article (unlimited access to the game) |
|  | Graves *et al.*^36^ | ++, article (intervention participants were given two jOG devices for home-use, Two devices were given to discourage sedentary play during multiplayer gaming, OG links a hip-worn pedometer to a standard console controller and encourages gamers to step on the spot to use directional controls to generate on-screen character movement in games) |
|  | Howie *et al.*^38^ | ++, article (During the intervention period, participants were provided with a PlayStation3 (Sony, Tokyo, Japan) with Move and Eye input devices, an Xbox360 (Microsoft, Redmond, USA) with Kinect input devices and a range of non-violent games to use at home for 16 weeks.) |
|  | Maddison *et al.*^35,39^ | ++, article (received an up-grade (hardware and games) of existing gaming technology that enabled them to play active video games at home. Sony PlayStation EyeToy (Sony) was used |
|  | Maloney *et al.*^42^ | ++, article (families in the intervention group were provided with all equipment necessary to play DDR in the home (PlayStation2 game console (Sony Corporation of America, New York, NY), DDR MAX2 game (Konami of America, Redwood City, New York) and two padded dance mats |

+ = present in all probability; ++ = present beyond all reasonable doubt. IG1 =intervention group 1; IG2 = intervention group

| **BCT** | **Study** | **Text coded** |
| --- | --- | --- |
| 12.5 Adding objects to the environment | Mark & Rhodes^24^ | Intervention (Exergame); ++, article (received a GameBike (Cat Eye Electronics Ltd., Boulder, Colorado) which is a stationary bicycle that reads both speed and steering allowing participants to play a number of games on a Sony PlayStation 2 (Sony Computer Entertainment America Inc, Foster City, California) console. If families did not own a PlayStation 2, they were loaned one by the research team. Three games were provided (Shrek Smash’N’Crash, ATV Off-road Fury, and Grand Turismo 3) |
|  |  | Control (Stationary bike); ++, article (received a GameBike without the interactive components installed, thereby acting as a traditional stationary bicycle) |
|  | Ni Mhurchu *et al.^23^* | ++, article (families of the intervention group received a Game-Bike (Cat Eye Electronics Ltd., Boulder, Colorado) which is a stationary bicycle that reads both speed and steering allowing participants to play a number of games on a Sony PlayStation 2 (Sony Computer Entertainment America Inc, Foster City, California) console.) |
|  | Rhodes *et al.*^40^ | Intervention (Exergame); ++, article (Participants in the Exergame group received a Hoggan Health interactive video gaming system linked to a SonyPlaystation3 and a television monitor) |
|  |  | Control (Stationary bike); ++, article (The comparison group received the Hoggan Health stationary bike without the videogame component and was instructed to exercise during each training session while watching TV.) |
|  | Rubin *et al.* ^45,46^ | ++, article ( the intervention consisted of providing parents and their children with the Active Play at Home curriculum, activity equipment(e.g., balls, hoops, hurdles, and cones) and active video game equipment (e.g., Nintendo Wii console, board, and game software) needed to engage in PA 4 dIwkj1for 24 wk) |
|  | Staiano *et al.*^47^ | ++, article (The present GameSquad trial used exergaming, Each participant randomised to GameSquad was provided a Kinect® and Xbox 360® gaming console (Microsoft, Redmond, WA), a 24-week Xbox Live subscription, and four exergames (Your Shape: Fitness Evolved 2012, Just Dance 3, Disneyland Adventures, and Kinect Sports Season 2), provided a Fitbit Zip (Fitbit, San Francisco, California) to wear during the 24-week period) |
|  | Tuominen *et al.*^48,49^ | ++, article (were instructed to use the movement to music video programme) |
| 13.1 Identification of self as role model | Rubin *et al.* ^45,46^ | ++, article (Further, self-efficacy in the children will be targeted through parents serving as skilled or learning models for the activity and providing verbal encouragement throughout the program) |

+ = present in all probability; ++ = present beyond all reasonable doubt

IG1 =intervention group 1; IG2 = intervention group
